# *MOCOS*‐associated renal syndrome in a Brown Swiss cattle

**DOI:** 10.1111/jvim.16856

**Published:** 2023-09-07

**Authors:** Joana G. P. Jacinto, Leonore Bettina Küchler, Laureen M. Peters, Elke Van der Vekens, Corinne Gurtner, Franz R. Seefried, Mireille Meylan, Cord Drögemüller

**Affiliations:** ^1^ Department of Veterinary Medical Sciences University of Bologna Ozzano Italy; ^2^ Institute of Genetics, Vetsuisse Faculty University of Bern Bern Switzerland; ^3^ Institute of Veterinary Pathology, Vetsuisse Faculty University of Bern Bern Switzerland; ^4^ Clinical Diagnostic Laboratory, Department of Clinical Veterinary Medicine, Vetsuisse Faculty University of Bern Bern Switzerland; ^5^ Division of Clinical Radiology, Department of Clinical Veterinary Medicine, Vetsuisse Faculty University of Bern Bern Switzerland; ^6^ Qualitas AG Zug Switzerland; ^7^ Clinic for Ruminants, Department of Clinical Veterinary Medicine, Vetsuisse Faculty University of Bern Bern Switzerland

**Keywords:** bovine, kidney disease, precision medicine, rare disease, urolithiasis, xanthine

## Abstract

**Background:**

A recessive form of *MOCOS*‐associated xanthinuria type II is described in Tyrolean grey cattle. A similar case was identified in a 5‐month‐old Brown Swiss calf with hoof overgrowth, rough coat, urine sediment, and pneumonia.

**Hypothesis/Objectives:**

To characterize the disease phenotype, to evaluate its genetic etiology, and to determine the prevalence of the deleterious allele in the Brown Swiss population.

**Animals:**

An affected calf, its parents, and 65 441 Swiss dairy cattle.

**Methods:**

The affected animal was clinically examined and necropsied. Microarray genotyping was used to determine the genotypes and to assess the frequency of the *MOCOS* allele in a Brown Swiss control cohort.

**Results:**

Ultrasonography revealed hyperechoic renal pyramids with multifocal distal shadowing and echogenic sediment in the urinary bladder. Necropsy revealed suppurative bronchopneumonia and urolithiasis. Histology revealed numerous nephroliths with multifocal chronic lymphohistiocytic interstitial infiltrates, fibrosis, tubular degeneration, chronic multifocal glomerulonephritis with sclerosis, and bilateral hydronephrosis. Dysplastic changes were observed in the corium of the claw and the cornea. Genetic testing identified the homozygous presence of a known *MOCOS* frameshift variant in the case. Both parents were heterozygous and the prevalence of carriers in genotyped Brown Swiss cattle was 1.4% (342/24337).

**Conclusions and Clinical Importance:**

The findings were consistent with the diagnosis of a recessive renal syndrome similar to xanthinuria type II described in Tyrolean grey cattle. The prevalence of the deleterious *MOCOS* allele is low in the Brown Swiss breed. However, mating of carriers should be avoided to prevent further losses.

**Abbreviations**
CBC: complete blood count
GLDH: glutamate dehydrogenase
LDH: lactate dehydrogenase
PBA: plasma biochemistry analysis
RI: reference intervals
T0: at age 5 months
T1: at age 7 months

## 1 INTRODUCTION

Sporadic cases of inherited renal disorders occur in domestic animals; to date, 3 recessively inherited disorders affecting the urinary system with known causal variants have been reported in cattle. One of these is the autosomal recessively inherited *SLC2A2*‐related Fanconi syndrome in Original Braunvieh and Fleckvieh cattle (OMIA000366‐9913).^1^, ^2^ This syndrome is clinically characterized by retarded growth, polyuria, polydipsia, glycosuria, as well as poor claw, horn, and coat quality. At necropsy, a pale renal cortex, renal hypoplasia, tubulonephrosis of the proximal tubules with protein and glucose‐rich contents are noticed.^1^, ^2^ In Japanese black cattle, recessive forms of renal dysplasia associated with 2 different harmful deletions in the *CLDN16* have been described (OMIA001135‐9913).^3^, ^4^ Clinically, affected animals show dullness, retarded growth, overgrowth of hooves; severe renal failure, and renal atrophy is present at necropsy.^3^, ^4^ Furthermore, recessive forms of xanthinuria type II associated with 2 independent deleterious variants in *MOCOS* have been reported in Tyrolean grey and Japanese black cattle (OMIA001819‐9913).^5^, ^6^ In analogy to renal dysplasia, affected animals show dullness, retarded growth, overgrowth of the hooves and severe renal failure. In contrast, animals show urolithiasis caused by xanthine calculi.^7^ This is because of the absence of the MOCOS enzyme that normally sulfurates the molybdenum cofactor of xanthine dehydrogenase and aldehyde oxidase, and is thus required for their enzymatic activities. On necropsy, affected animals display renal atrophy and dysplasia, as well as xanthinuric urolithiasis.^7^

Our aim was to describe the phenotype of a Brown Swiss calf affected by a renal syndrome, to identify its genetic etiology and to estimate the prevalence of the underlying *MOCOS* variant in Brown Swiss cattle.

## 2 MATERIALS AND METHODS

See Supporting Information.

## 3 RESULTS

### 3.1 Clinical presentation

**FIGURE 1:**
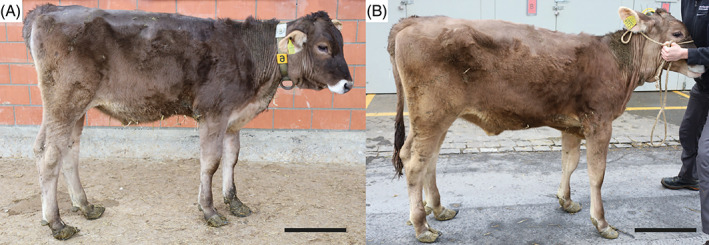
Brown Swiss bovine affected by renal syndrome. (A) Affected calf at the age of 5 months. Note dullness, poor nutritional condition, rough haircoat, and claw overgrowth. (B) Affected heifer at the age of 7 months. Note the worsening of the general condition and lack of evident growth in comparison to the age of 5 months. Bar = 25 cm.

**FIGURE 2:**
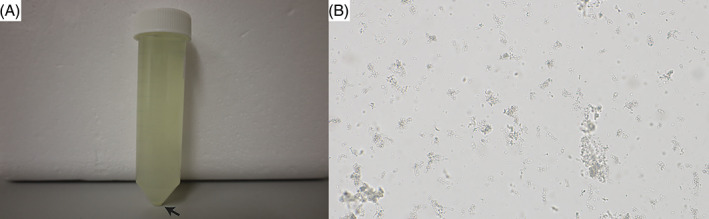
Urinary analysis of the Brown Swiss heifer affected by renal syndrome. (A) Macroscopic urine analysis. Note that the urine is turbid with sediment (arrow). (B) Microscopic sediment analysis revealing high numbers of colorless to slightly brown, amorphous crystals (100× magnification).

At T0, the calf showed dullness, retarded growth and poor nutritional status associated with muscular atrophy (Figure 1A). The oculoconjunctival mucous membranes were congested and the episcleral vessels were injected. The calf showed overgrowth of the hooves, more severe in the hind than in the front limbs, associated with gait difficulties. The respiratory rate was slightly elevated with 42 respirations/min; the calf was 5% dehydrated. Lung auscultation revealed increased expiratory sounds mainly in the cranioventral lobes. Notably, upon transabdominal palpation of the right kidney, the animal showed signs of pain: it looked to the right flank, grinded its teeth, and vocalized. Spontaneously voided urine was macroscopically pale yellow, and turbid with sediment (Figure 2A). The urine specific gravity was 1.010, the pH 7.6 and urine dipstick analysis showed marked hematuria (+++, corresponding to approximately 50 erythrocytes/μL). Microscopic sediment analysis revealed the presence of occasional erythrocytes, low numbers squamous epithelial and urothelial cells, as well as high numbers of colorless to slightly brown, amorphous crystals (Figure 2B). Complete blood count showed moderate erythrocytosis (9.72 × 10^12^/L; reference intervals [RI] 5‐7.2), with microcytosis (mean cell volume 30 fl; RI 38‐51) and increased red cell distribution width (22.4%; RI 15‐19.4), but with normal hematocrit (29%; RI 24‐35) and mean cell hemoglobin concentration (351 g/L; RI 340‐380). There was a mild monocytosis (1.24 × 10^9^/L; RI 0‐0.33), confirmed by a manual differential leukocyte count. On PBA a marked azotemia (creatinine 5.24 mg/dL; RI 0.97‐1.47; urea 74 mg/dL; RI 4.2‐20.4), a mild hyperkalemia (5.98 mmol/L; RI 3.9‐5.75), and mildly increased activities of glutamate dehydrogenase (GLDH; 32 U/L; RI 0‐17) and lactate dehydrogenase (LDH; 1675 U/L; RI 876‐1262) were observed.

**FIGURE 3:**
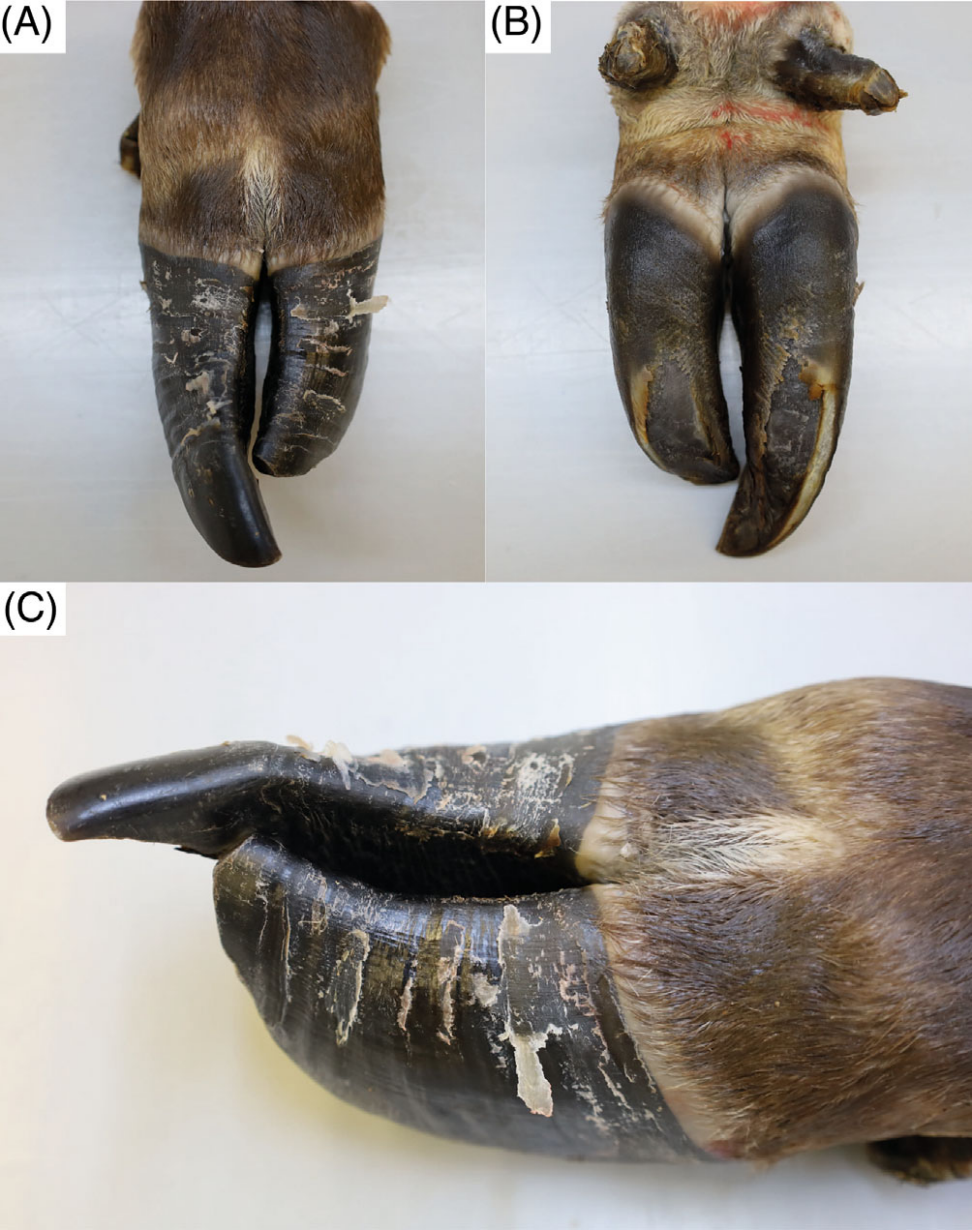
Hoof overgrowth in a Brown Swiss heifer affected by renal syndrome at 7 months of age. (A‐C) Dorsal view of the left hind hooves. Note that the wall horn of the hooves is brittle, with parallel fissures to the coronary band. Note that the medial claw is longer than the lateral.

**FIGURE 4:**
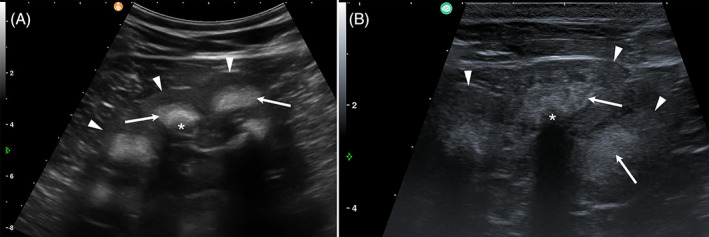
Transcutaneous (A) and transrectal (B) ultrasonographic images of the right and left kidneys, respectively, in a Brown Swiss heifer affected by renal syndrome. Bilaterally renal pyramids (arrows) are hyperechoic to the renal cortex (arrowheads). Within these pyramids, curved hyperechoic areas with distal acoustic shadowing (*) were visible in nearly all renal lobes. These areas appeared to outline the transition to the apices of the pyramids and represent the sandy uroliths observed on necropsy. Cranial is to the left in both images.

At T1, physical examination revealed similar clinical findings as upon first clinical examination but with worsening of the general condition (Figure 1B). The coat was dull with areas of hypotrichosis mainly at the base of the ears, on the neck and rump. Hoof overgrowth was more pronounced (Figure 1B). In addition, the wall horn of the hooves was brittle, with fissures parallel to the coronary band. In particular, the hind hooves displayed crossing of the tips because of the increased length (Figure 3). The heifer also showed bilateral serous ocular discharge and spontaneous dry cough. Auscultation of the lungs revealed increased inspiratory and expiratory sounds mainly in the cranioventral thorax. The animal continued to show pain upon palpation of the right kidney. On transabdominal ultrasonography, the right kidney had a normal shape, but the renal pyramids were hyperechoic to the cortex. Within these pyramids, curved hyperechoic areas with distal acoustic shadowing were visible in nearly all renal lobes. These areas appeared to outline the transition to the apices of the pyramids (Figure 4A). Transrectal ultrasonography of the left kidney that revealed similar findings (Figure 4B). The urinary bladder contained a moderate amount of gravity dependent sediment, hyperechoic to the adjacent urinary bladder wall. Analysis of spontaneously voided, pale pink and turbid urine was similar to previous, except that the hematuria was more severe (++++, or approx. 250 erythrocytes/μL on dipstick, with erythrocytes too numerous to count in the sediment), but no overt crystalluria. The erythrogram was similar at T1 compared to T0, and there was a neutrophilia (6.78 × 10^9^/L; RI 1‐3.5) with mild left shift (0.3 × 10^9^/L; RI <0.2) but no toxicity on microscopic blood smear examination, a continued monocytosis (0.72 × 10^9^/L) and a borderline lymphopenia (2.36 × 10^9^/L; RI 2.5‐5.5). The azotemia had mildly worsened (creatinine 5.42 mg/dL; RI 0.97‐1.47, urea 89.6 mg/dL; RI 4.2‐20.4), as had the increase in GLDH (56 U/L; RI 0‐17) and LDH (1823 U/L; RI 876‐1262) activity.

### 3.2 Pathology

**FIGURE 5:**
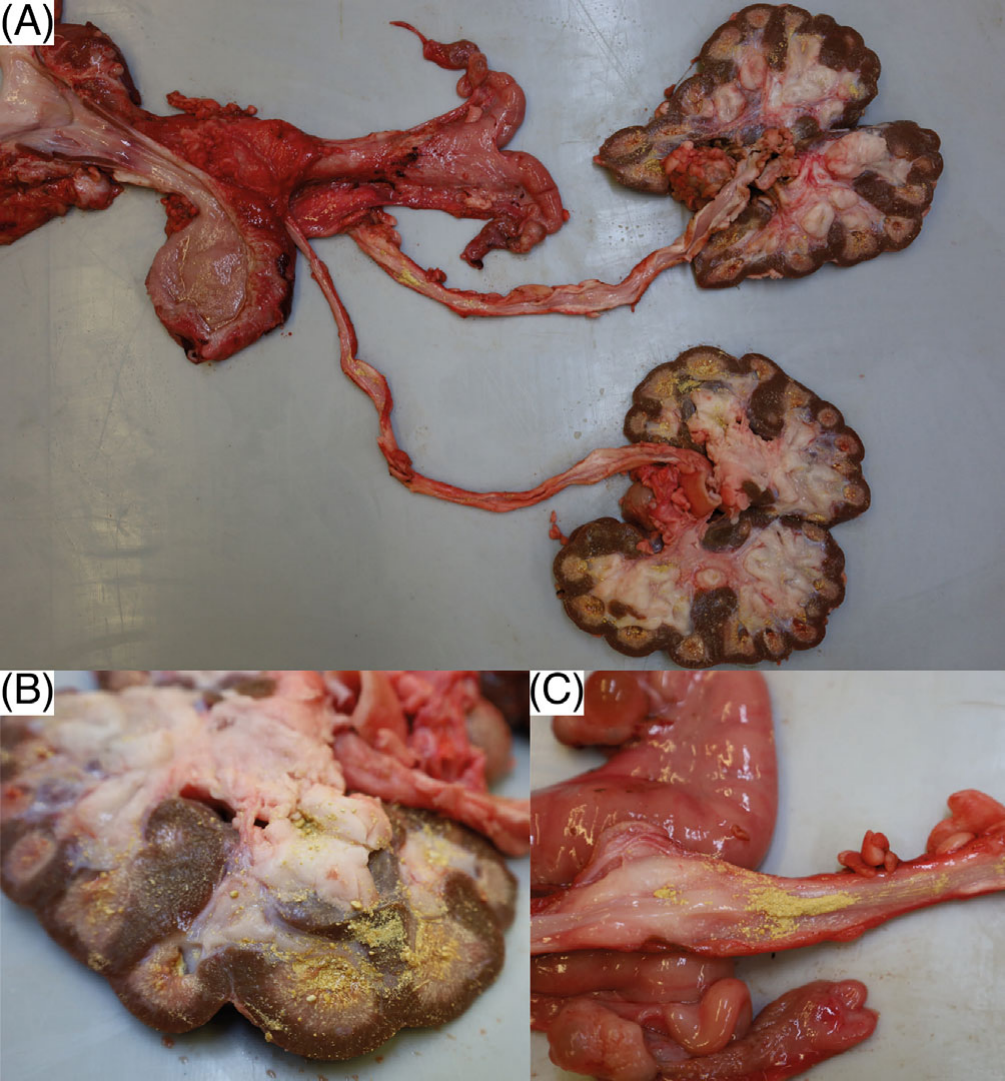
Gross pathology findings of the urinary system in a Brown Swiss heifer affected by renal syndrome. (A) Note uroliths in the kidneys, ureters and urinary bladder. (B) Note the renal calices diffusely filled with yellow, sandy material. The medulla appears diffusely moderately atrophied. The cortex and medulla are diffusely pale. (C) Note the ureter, diffusely filled with yellow, sandy material. Multifocally, there is a transmural irregular thickening of the wall.

Macroscopically, the horn on all claws and dewclaws was markedly overgrown, brittle, with prominent undulations. The horn of the dewclaws was easily detached from the laminar corium by slight traction. The distal trachea was filled with white foam mixed with yellow and turbid fluid. The tips of the cranial and middle pulmonary lobes contained multifocal to confluent severely pus‐filled areas. The bronchi and bronchioles in these areas were filled with exudate and dilated. The large intestine was moderately filled with brownish, pasty content. Several nematodes with thin flagella, about 2 cm long and 0.2 cm in diameter, adhered to the mucosa, compatible with *Trichuris* spp. Both kidneys appeared irregular in outline and shrunken. Both medulla and cortex were moderately atrophied, pale, and renal calices were diffusely filled with yellow, sandy material of <0.1 to 0.2 cm in diameter (Figure 5A,B). A mild to moderate amount of yellow sandy material, similar to that in the calicles, was present in the ureteral lumen (Figure 5C), and both ureters were moderately dilated. There was a multifocal transmural irregular thickening of the wall. Approximately 20 mL of slightly cloudy, yellowish urine with sandy material, analogous to that in the kidney and ureter, was present in the urinary bladder. Small amounts of the yellow sand adhered diffusely to the mucosa. The urethra was empty with an inconspicuous mucosa. Multifocal adherence of the yellow sandy material to the haired skin around the vulva was observed.

**FIGURE 6:**
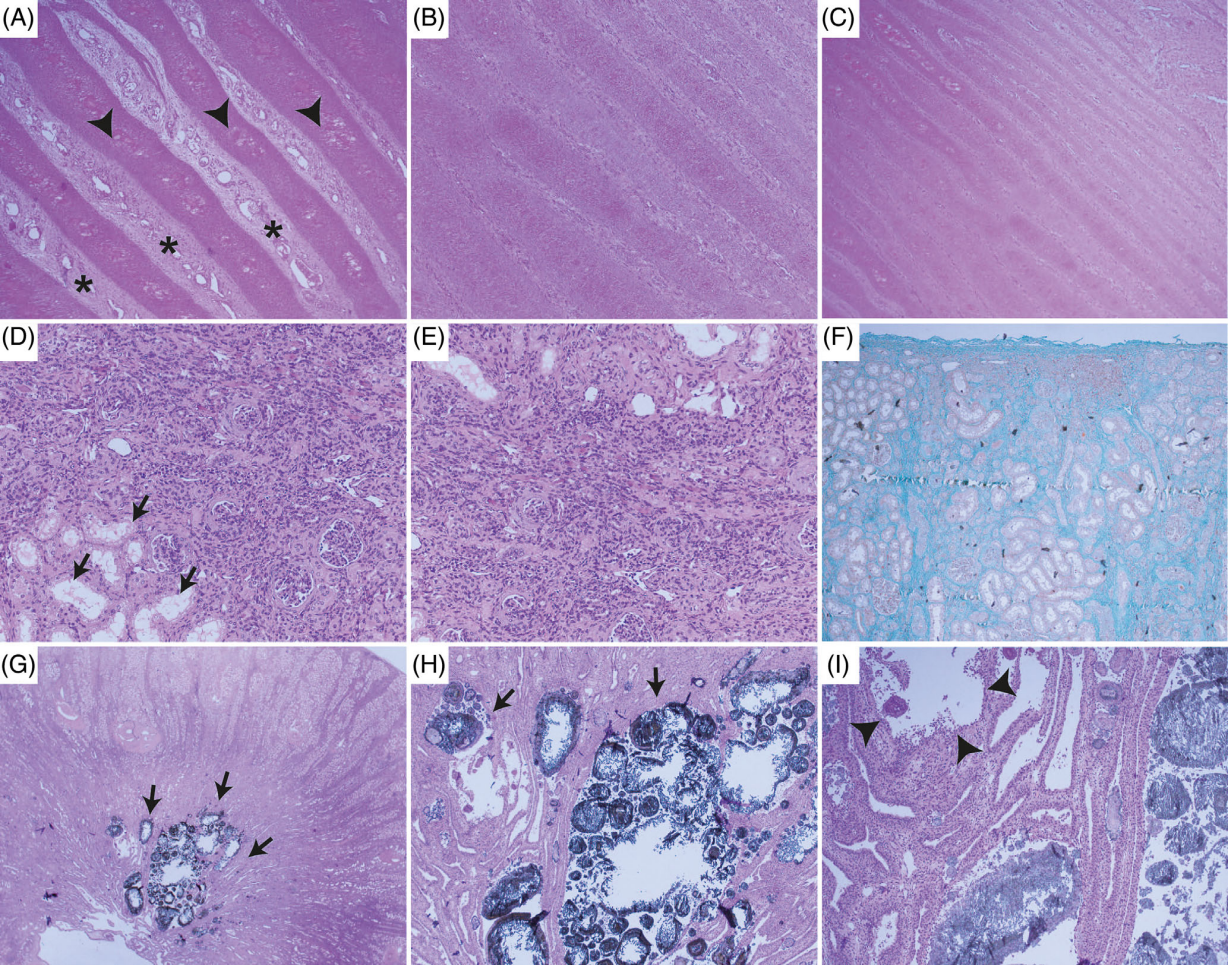
Histologic findings in a Brown Swiss heifer affected by renal syndrome. (A‐C) Hoof plantar dermis and epidermis, HE stain, 4× magnification. Hoof lamellae are irregular, differ in length and width, and are often plump. Keratinocytes arrangement is disorganized (epidermal dysplasia), ballooning degeneration and spongiosis is commonly observed (arrow head). The lamina propria is edematous and vessels are often markedly dilate (asterisk). (D and E) Kidney, cortex, HE stain, 20× magnification. The tissue architecture of the cortex is severely altered. In the interstitium, there is a large amount of eosinophilic, fibrillar extracellular material (collagen) surrounding and compressing the tubules, admixed with small numbers of predominantly lymphocytes. The lumina of the tubules are partially filled with homogeneous eosinophilic material (protein casts). Other tubules are dilated with degeneration of tubular epithelial cells (arrow). Glomeruli show thickening of the Bowman's capsule by fibrosis and hyperplasia of the parietal layer, formation of synechia, a mild increase in mesangial cells, atrophy, and glomerulosclerosis. Few glomeruli are fetal with small cells on the outskirts of the glomerular capillaries. (F) Kidney, Masson Trichrom stain, 2× magnification. The extracellular fibrillar material in the interstitium stains green in Masson Trichrome special staining, confirming the presence of collagen (fibrosis). (G) Kidney, uroliths, HE stain, 2× magnification. Numerous uroliths of up 500 μm in diameter are present predominantly within more distal tubules, leading to a severe dilatation (arrow). (H and I) Kidney, uroliths, HE stain, 4× and 10× magnification, respectively. Uroliths are composed of a center of eosinophilic to amphophilic material, surrounded by concentric, amorphous eosinophilic, irregular material, which is usually mineralized leading to a severe dilatation (arrow). Multifocally, macrophages and multinucleated giant cells surrounded the stones. Tubular epithelial cells undergo attenuation, necrosis, and/or vacuolar degeneration (arrow head). Attempts of regeneration with piling up of hypertrophied cells and mitotic figures can also be seen. Admixed with the uroliths are sloughed epithelial cells, proteinaceous material, and few lymphocytes and neutrophils.

Histologically, the primary and secondary dermal lamellae of the claws showed a highly irregular arrangement (Figure 6A‐C). They differed in length and width, some of which were rather long and slender and slightly tortuous, others appeared plump. Numerous blood vessels of different size, often irregularly dilated, others congested, and small areas of hemorrhages could be observed. The keratinized epithelium covering the lamellae was markedly hypertrophic with disorganized arrangement of epithelium, irregular keratinization, and multifocal ballooning degeneration (intracellular edema) and spongiosis (intercellular edema). Overall, these changes were compatible with a diffuse dermal and epidermal dysplasia of the claws.

All levels of the nephron and interstitium showed marked changes: glomeruli were of variable sizes, often shrunken with segmental to diffuse thickening of the basement membrane, multifocal sclerosis, a moderate increase in mesangium, marked thickening of the Bowman's capsule and multifocal synechia formation (Figure 6D‐F). Few neutrophils were trapped within the glomerular loops. Tubular lumina contained numerous uroliths of up to 500 μm in diameter, leading to severe dilatation, which was most pronounced in the collecting ducts. Uroliths consisted of an amorphous eosinophilic center, surrounded by golden‐brown, radiating, crystalline material that was multifocally mineralized (Figure 6G‐I). Smaller crystals were present in more cortical tubules. Tubular epithelium often contained crystalline to amorphous material as well, and many macrophages and multinucleate giant cells of Langerhans type surrounded these areas. The epithelium showed a variation of attenuation with flattened cells, degeneration, necrosis and/or loss. Attempts of tubular regeneration with piling up of hypertrophied cells and mitotic figures was observed. Besides the uroliths, tubular lumina multifocally contained eosinophilic homogeneous material (protein casts) and/or sloughed epithelium, cell debris, few lymphocytes and neutrophils. The basement membrane was often markedly thickened.

The renal interstitium showed a moderate to severe fibrosis, characterized by radiating deposition of extracellular, fibrillar material, which stained positive on Masson's Trichrome special stain and which showed birefringence under polarized light when stained with Congo red. Within the fibrous stroma were multifocal aggregates of predominantly lymphocytic inflammatory infiltrates and lesser numbers of macrophages. The calices appeared dilate with few uroliths as described above adhering to the urothelium, mild mucosal edema and similar inflammatory infiltrates and fibrosis as described for the interstitium.

Overall, the renal changes were consistent with a (a) severe multifocal chronic nephrolithiasis with severe lymphocytic interstitial nephritis and fibrosis, (b) a moderate multifocal chronic membranoproliferative glomerulonephritis with sclerosis, and (c) moderate bilateral hydronephrosis.

In the ureter, the epithelium was eroded or ulcerated, with few uroliths of similar in appearance to those described for the kidney adherent to the mucosa, and very few mixed inflammatory cells within the lamina propria. In the urinary bladder, the transitional epithelium was hyperplastic with formation of papillary projections, moderate submucosal edema, and a mild lymphocytic and plasmacellular inflammatory infiltrate. Multifocal uroliths were adherent to the mucosa.

These findings were consistent with a moderate chronic ureteritis with urolithiasis and hydroureter and a moderate diffuse lymphoplasmacytic and hyperplastic cystitis with urolithiasis.

Histologic examination of the lung tissue confirmed the presence of chronic high‐grade suppurative bronchopneumonia with bronchiectasis.

The clinicopathological findings were consisted with xanthinuria‐like renal syndrome type II and unrelated bronchopneumonia.

### 3.3 Genetic analysis

We hypothesized that the presented case of xanthinuria‐like renal syndrome type II might be explained by a rare recessively inherited variant. The affected heifer was homozygous for the known *MOCOS* frameshift variant (NP_776506.1:p.Ser628ValfsTer9) and both parents were confirmed as heterozygous carriers of this deleterious allele. No other *MOCOS* homozygotes were observed in 24 337 Brown Swiss cattle (Table 1). Evaluation of the prevalence of this deleterious allele in Swiss dairy cattle, including Brown Swiss, Original Braunvieh, Grauvieh, Simmental, Swiss Fleckvieh, and Holstein, revealed a frequency of 0.7% in Brown Swiss and in Grauvieh, 0.2% in Original Braunvieh, and 0% in other breeds (Table 1).

**TABLE 1:** Occurrence of the deleterious *MOCOS* allele in Swiss dairy cattle.

	*MOCOS* genotype	
	Ref/ref	Ref/var
Brown Swiss	23 995	342^a^
Original Braunvieh	4085	19
Grauvieh	806	8
Simmental	3926	0
Swiss Fleckvieh	4035	0
Holstein	28 227	0
Total	65 074	369

^a^ Including both parents.

## 4 DISCUSSION

Extensive clinical, pathological, and genetic studies of a Brown Swiss heifer with a renal syndrome resembling *MOCOS*‐related xanthinuria type II (and unrelated bronchopneumonia) revealed an inherited cause for the observed phenotype. A possible genetic origin was evaluated using a custom genotyping array as a diagnostic tool covering many known causal variants. This confirmed the putative genetic cause of this disease, which was previously unknown in Brown Swiss cattle.

The clinical findings in the affected animal were very similar to the *MOCOS*‐related xanthinuria type II cases in Tyrolean grey and Japanese black cattle.^5^, ^6^ These affected cattle showed dullness, retarded growth, hooves overgrowth, and renal disease.^6^, ^7^ As in the case presented here, affected Tyrolean grey cattle had azotemia consistent with renal disease. In addition, in our case, mild increase of plasma hepatic enzymes was noticed, suggesting mild hepatocellular injury. Urolith analysis using Fourier transform infrared spectroscopy in affected Tyrolean grey cattle demonstrated the presence of xanthine crystals.^5^ In our case, we identified urinary crystals, which, based on light microscopy, could have been classified as either amorphous urates (in case of acidic pH), amorphous phosphates (in alkaline pH), or xanthine (in acid or neutral pH).^8^, ^9^ Considering that the urine pH was neutral, the latter is the most likely scenario.

Differences to previously described cases were found at necropsy. The Brown Swiss heifer did not show renal tubular dysplasia, in contrast to previous reports in Tyrolean grey and Japanese black cattle.^5^, ^6^ In our case, the analysis of tissues of the urinary system revealed lesions compatible with the sequela of urolithiasis, specifically dilatation of the renal tubules and ureters with intra‐lesional stones, epithelial damage, inflammation, edema and interstitial fibrosis. In addition, the glomeruli also showed moderate inflammation and sclerosis. Therefore, the changes in the kidney appear to be related to massive crystal formation in the kidney, most likely because of xanthine uroliths.

Our report describes a homozygous *MOCOS* mutant case associated with renal syndrome in Brown Swiss cattle, confirming an identical genetic etiology for a disorder known to exist in Tyrolean grey cattle.^5^ Thus, this report provides an example of a recessive hereditary defect that occurs across breeds as previously observed in other recessively inherited diseases in cattle.^1^, ^2^, ^10^, ^11^ It is possible that the origin of this rare disease‐causing *MOCOS* variant in the Brown Swiss breed is because of either accidental crossbreeding or targeted introgression of Tyrolean grey cattle, a closely related Alpine breed known as Grauvieh in Switzerland. Alternatively, it may indicate a very ancient origin of the derived allele, before the formation of these modern breeds. These breeds originate from the same geographical area and share a significant genetic relationship.^12^, ^13^ Interestingly, some carriers of the *MOCOS* variant have also been observed in the founding breed of Brown Swiss cattle that originated in North America from animals bred in Switzerland, the so‐called Original Braunvieh population. The Original Braunvieh breed is the ancestor of the modern global Brown Swiss population, which was created in the USA from animals purchased in Switzerland between 1869 and 1910.^14^ Since the 1960s in Switzerland, Original Braunvieh cows have been crossed with American Brown Swiss bulls using artificial insemination, resulting in today's Brown Swiss population.

The purpose of this report is to alert veterinarians and cattle breeders to the possible future occurrence of a renal syndrome in Brown Swiss cattle, although the prevalence of the pathogenic *MOCOS* allele is relatively low. Genetic testing provides an accurate diagnosis. Carrier mating can be avoided in the future to prevent further affected calves.

## CONFLICT OF INTEREST DECLARATION

Authors declare no conflict of interest.

## OFF‐LABEL ANTIMICROBIAL DECLARATION

Authors declare no off‐label use of antimicrobials.

## INSTITUTIONAL ANIMAL CARE AND USE COMMITTEE (IACUC) OR OTHER APPROVAL DECLARATION

The Cantonal Committee for Animal Experiments approved the collection of blood samples from control cattle that were used in this study (Canton of Bern; permit BE 71/19).

## HUMAN ETHICS APPROVAL DECLARATION

Authors declare human ethics approval was not needed for this study.

## Supporting information


**Data S1:** Supporting Information.
