# Supplementary material for: MOCOS‐associated renal syndrome in a Brown Swiss cattle
Source: J Vet Intern Med. 2023 Sep 7;37(6):2603–9. doi: 10.1111/jvim.16856 (PMC10658536; doi:10.1111/jvim.16856)
Supplement: Supplementary file 1 — Data S1: Supporting Information. [file JVIM-37-2603-s001.pdf]

Clinicopathological investigation

A 5-months-old Brown Swiss calf was referred to the Clinic for Ruminants of University of Bern because of hoof overgrowth and failure to thrive. In the first month of life, the calf had a clinical history of weakness, dullness and hair loss. The affected calf was clinically examined, and a complete blood count (CBC), plasma biochemical analysis (PBA) and urinalysis were obtained at age of 5 months (T0) and repeated 2 months later (age 7 months; T1). Additionally, at T1, urinary system ultrasonography was performed. The heifer was euthanized at T1 due to severe emaciation, lameness, bronchopneumonia and renal disease, and was sent for necropsy. Samples were collected, fixed in 10% buffered formalin, embedded in paraffin, and processed for histological examination. Five-micron histological sections specimens were stained with hematoxylin and eosin, Masson`s Trichrome special stain (collagen) and Congo red stain.

Genetic investigation

Desoxyribonucleic acid was extracted from an EDTA-blood sample from the heifer, as well as from EDTA-blood from the dam and semen from the sire. The three subjects were genotyped for the NP_776506.1: p.Ser628ValfsTer9 frameshift variant in *MOCOS*, previously associated with renal syndrome (xanthinuria type II) in Tyrolean grey cattle[5], with a custom genotyping array, using Axiom Microarray Genotyping Technology (SWISScow). The array was been developed under the umbrella of the Swiss routine genomic system, which has genotyped several thousand animals from current Swiss dairy population since 2020 and contains 62,642 variants, including common "routine markers" considered for genomic selection, as well as 128 known bovine variants that cause bovine Mendelian disorders. Evaluation of the prevalence of this deleterious allele in Swiss dairy cattle, including Brown Swiss, Original Braunvieh, Tyrolean grey Simmental, Swiss Fleckvieh, and Holstein was performed using a total of 65,443 cattle. Unfortunately, the array did not include the NP_776506.1: p.Tyr257del variant in *MOCOS*, previously associated with the renal syndrome in Black Japanese cattle[6].
